# Development and implementation of a Dependable, Simple, and Cost-effective (DSC), open-source running wheel in High Drinking in the Dark and Heterogeneous Stock/Northport mice

**DOI:** 10.3389/fnbeh.2023.1321349

**Published:** 2024-01-15

**Authors:** Kolter Grigsby, Zaynah Usmani, Justin Anderson, Angela Ozburn

**Affiliations:** ^1^Portland Veterans Affairs Medical Center, Research and Development Service, Portland, OR, United States; ^2^Department of Behavioral Neuroscience, Oregon Health and Science University, Portland, OR, United States

**Keywords:** physical activity, wheel-running, alcohol, genetic animal models, reinforcement

## Abstract

Maintaining healthy and consistent levels of physical activity (PA) is a clinically proven and low-cost means of reducing the onset of several chronic diseases and may provide an excellent strategy for managing mental health and related outcomes. Wheel-running (WR) is a well-characterized rodent model of voluntary PA; however, its use in biomedical research is limited by economical and methodical constraints. Here, we showcase the DSC (Dependable, Simple, Cost-effective), open-source running wheel by characterizing 24-h running patterns in two genetically unique mouse lines: inbred High Drinking in the Dark line 1 [iHDID-1; selectively bred to drink alcohol to intoxication (and then inbred to maintain phenotype)] and Heterogeneous Stock/Northport (HS/Npt; the genetically heterogeneous founders of iHDID mice). Running distance (km/day), duration (active minutes/day) and speed (km/hour) at 13-days (acute WR; Experiment 1) and 28-days (chronic WR; Experiment 2) were comparable to other mouse strains, suggesting the DSC-wheel reliably captures murine WR behavior. Analysis of 24-h running distance supports previous findings, wherein iHDID-1 mice tend to run less than HS/Npt mice in the early hours of the dark phase and more than HS/Npt in the late hours of dark phase/early light phase. Moreover, circadian actograms were generated to highlight the broad application of our wheel design across disciplines. Overall, the present findings demonstrate the ability of the DSC-wheel to function as a high-throughput and precise tool to comprehensively measure WR behaviors in mice.

## 1 Introduction

Human sedentary behavior accounts for more than 40 known chronic diseases and conditions, many of which are seen in high frequency (i.e., heart disease and diabetes) and play an undeniable role in rising health care costs, worldwide (Lightfoot et al., 2018; Ruegsegger and Booth, 2018). Modest estimates predict that physical inactivity costs the U.S. approximately $507 billion in health care expenses annually (Chenoweth and Leutzinger, 2006). Although public health efforts to incorporate more daily physical activity are growing (EU Working Group “Sport Health”, 2008; World Health Organization, 2010; Brown et al., 2012; 2018 Physical Activity Guidelines Advisory Committee, 2018), there remains a lack of understanding of the molecular and behavioral effects of physical inactivity on our daily health and well-being. This is especially true for understanding the role of daily physical activity (PA) in regulating other patterned behaviors, such as harmful alcohol and substance use.

Today's low societal valuation of PA stems from what Lieberman describes as a “cultural dysevolution”, wherein the advent of technology (namely sophisticated transportation systems) and a health care system that favors pharmacotherapies for treating sedentary-related diseases over promoting PA has resulted in an inactivity epidemic that is antithetical to our natural history (Lieberman, 2015). Moreover, preclinical research applies the same sedentary framework across biomedical science—in which “control conditions” for disease models are widely accepted to be sedentary. This is compounded by the high initial cost of commercial PA equipment and software. In response, we have developed a highly adaptable and inexpensive, open-source mouse running wheel with continual recording as a means of lowering the financial barrier to study PA in rodents—the Dependable, Simple, and Cost-effective (DSC) running wheel. We expect these wheels to have broad implementation across biomedical research, particularly in addiction and circadian-related research.

Voluntary wheel-running has long been studied in captive animals, with reports in rodents dating back to 1898 (Sherwin, 1998). Meijer and Robbers further demonstrated that wild mice will voluntarily run on outdoor running wheels at levels that are comparable to common inbred mouse strains, such as C57BL/6J (Meijer and Robbers, 2014). These results establish voluntary wheel-running as a naturally occurring model of PA for mice. Moreover, wheel-running reflects human physical activity in having notable individual variability (Swallow et al., 1998). Findings as early as 1933 have noted strain differences in spontaneous PA behavior for both rats (Rundquist, 1933) and mice (Friedman et al., 1992; Dohm et al., 1994), suggesting a key genetic component underlying PA and exercise capacity. Using a well-known model of conditioned reward evaluation in rodents, Greenwood et al. demonstrated that male rats develop a preference for contexts associated with wheel-running at ~6-weeks, but not 2-weeks (Greenwood et al., 2011). Similarly, operant testing in both male mice (Belke and Garland, 2007) and rats (Belke and Pierce, 2015) show that rodents are positively motivated to gain access to running wheels. Taken together, wheel-running represents a translationally relevant model of PA that is completely voluntary, naturalistic, and behaviorally reinforcing. The primary goal of the present work is to promote the widespread adoption of wheel-running (across all biomedical research, but particularly addiction and circadian research) by lowering the economic and technological barriers for access to 24-h recording of voluntary PA in mice. To achieve this goal, the present work highlights the development and implementation of the DSC-wheel for others to adopt.

Like other natural and drug rewards (e.g., ethanol drinking), consistent PA creates physiological remodeling and neural adaptations across the mesocorticolimbic system—the primary neural regulator of reward and motivation (Knab and Lightfoot, 2010). PA also increases striatal dopamine (Freed and Yamamoto, 1985; Roberts et al., 2012), as well as endogenous plasma and brain levels of opioids and endocannabinoids, all of which positively influence reward and reinforcement (Schwarz and Kindermann, 1992; Chaouloff et al., 2011). Due to their similarities in restructuring the reward system, there is growing interest in the use of PA as an adjunct treatment for substance and alcohol use disorders [e.g., NOT-AA-21-015; Notice of National Institute of Alcohol Abuse and Alcoholism (NIAAA) participation in “Testing Interventions for Health-Enhancing Physical Activity”]. Using Preferred Reporting Items Systematic Reviews and Meta-Analysis (PRISMA) guidelines to address the efficacy of PA to treat and improve symptomologies associated with addiction, Patterson et al. (2022) found that nearly 75% of the included studies identified a marked improvement in addiction-related outcomes as a result of PA, clearly demonstrating the importance of incorporating PA as an adjunctive treatment option for harmful substance use (Patterson et al., 2022).

The primary goal of the present work is to showcase the capacity of the DSC-wheel to track 24-h home cage running in mice. Prior work has characterized circadian running patterns of male High Drinking in the Dark line 1 (HDID-1) mice [which were selectively bred to drink to intoxicating blood alcohol levels (BALs) and represent a unique genetic risk model for harmful drinking], as well as their genetically heterogeneous founders, Heterogeneous Stock/Northport (HS/Npt) mice. Analysis of circadian waveforms revealed that HDID-1 mice displayed fewer wheel turns than HS/Npt mice in the first half of the dark phase (~2–8 h after lights off) but had greater activity in the early hours of the light cycle (~2–4 h after lights on; McCulley et al., 2013). Although a critical first step in the evaluation of genetic risk for harmful drinking and circadian activity patterns, these studies were conducted in sound attenuated chambers (i.e., no visual, auditory, or olfactory cues between mice), evaluated a single time point (3-weeks of wheel access), and only tested male mice. Here, we have the unique opportunity to assess whether these phenotypic differences extend to home cage running characteristics in female and male inbred HDID-1 (iHDID-1) mice and HS/Npt mice at two critical stages of PA reinforcement—acute (which is physiologically stressful) and chronic wheel-running (which is behaviorally reinforcing; Fediuc et al., 2006; Greenwood et al., 2011). The present work demonstrates the utility and reliability of DSC running wheels by 1) characterizing 24-hr home cage PA patterns of female and male inbred HDID-1 (iHDID-1) and HS/Npt mice at acute (13-days) and chronic (28-days) timepoints across the following parameters: distance, running duration, and speed, and 2) providing granular data as visualized by circadian actograms and density plots.

## 2 Materials and methods

### 2.1 Animals

Adult male and female inbred High Drinking in the Dark (iHDID-1; S26.F28) and Heterogeneous Stock/Northport (HS/Npt; G104R5) mice were used for all experiments. Experiments were split up among two cohorts for acute and chronic wheel-running (*n* = 39/cohort; 9–10/sex/genotype/cohort). HDID-1 mice were initially selectively bred to drink alcohol to intoxication and then inbred at S25 to maintain this phenotype (Crabbe et al., 2009, 2019). HS/Npt mice are the genetically diverse founders of the iHDID-1 line and were derived from an 8-way intercross consisting of the following inbred strains from Jackson Laboratory (Bar Harbor, ME): A/J; AKR/J; BALB/cJ; C3H/HeJ; C57CL/6J; CBA/J; DBA/2J; LP/J (Hitzemann et al., 1994; Crabbe et al., 2009). Importantly, HS/Npt account for ~36% of the total *Mus musculus* genetic variation (Roberts et al., 2007). The iHDID-1 and HS/Npt colonies are maintained within the VA Portland Healthcare System Veterinary Medical Unit, where mice are group-housed, and bred and maintained on a reverse 12-h light/dark cycle with lights off at 7:30 am [Pacific Standard Time (PST)]. Animals were moved to their behavioral testing room one week prior to behavioral testing [on postnatal days 42–56 (acute WR) and 78–150 (chronic WR)] to habituate them to individual housing, new sipper tubes, and a shifted light/dark cycle (10:20 lights off, 22:20 lights on PST). Mice had *ad libitum* access to water and Purina 5LOD chow (PMI Nutrition International, Brentwood, MO, USA), and were housed on Bed-o' cobs^®^ bedding (The Andersons, Inc., Maumee, OH, USA) in standard polycarbonate cages with stainless steel wire tops. All procedures were approved by the VA Portland Health Care System Animal Care and Use Committee and were conducted in accordance with NIH Guidelines for the Care and Use of Laboratory Animals.

### 2.2 DSC-wheel design

**Figure 1:**
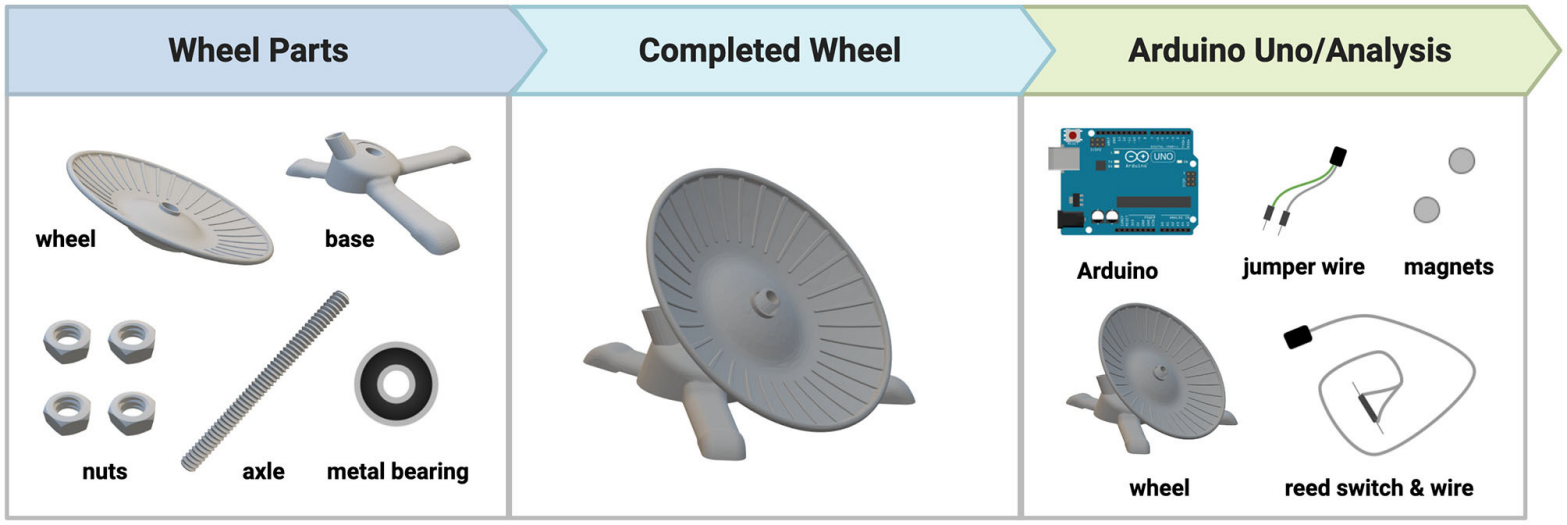
DSC-Wheel design and parts.

As illustrated in Figure 1, the DSC-wheel apparatus consists of four parts: wheel, base, nut, and axle (all of which are completely customizable). The following measurements (length; width; height) were used to fit a standard shoebox mouse cage: Wheel (127 mm; 127 mm; 23 mm); Base (131 mm; 140 mm; 37 mm); Nut (16.63 mm; 16.63 mm; 7.20 mm); Axle (10 mm; 10 mm; 120 mm). The DSC-wheel was designed on the open-source 3D modeling software, Tinkercad (Autodesk, San Rafael, CA) and sliced for 3D printing using Cura (Version 4.12.1; Ultimaker, Utrecht, Netherlands). All parts were printed using polyethylene terephthalate glycol (PETG) filament on an Ender-3 3D Printer (Creality 3D, Shenzhen, China). For the experiments below, 50 wheels, bases, and axles were printed, and 200 nuts were printed (4 per running wheel). In total, 1 DSC running wheel required 144 g of PETG and took 7 h and 57 min to print (using a 1 mm nozzle for the wheel and base and a 0.6 mm nozzle for the nuts and axle), one wheel used 96 g of PETG and took 4 hrs and 20 min to print, one base used 38 g of PETG and took 1 h and 45 min to print, one set of 4 nuts used 4g of PETG and took 28 min to print, and one axle used 6 g of PETG and took 1 h and 24 min to print. Metal bearings (10 mm) were inserted into the back of the wheel to allow for ease of rotation. Refer to Figure 1 for DSC-wheel components and design. To measure wheel-running behaviors, a magnet was attached to the back of each running wheel, and wheel rotations were recorded using a magnetic reed switch connected to the Arduino Uno (Arduino, New York, New York) open-source microcontroller board. Data was recorded for 24 h a day (in 1-min bins) for the duration of the study using the Adafruit Data Logger Shield (Adafruit, Brooklyn, New York). Table 1 includes a list of all materials needed to build the DSC-wheels (*n* = 50) and data loggers (*n* = 3) used in this work. Another key feature of the DSC-wheel is their ability to lock, whereby tightening the nut above the wheel prevents rotation. As noted by Dubreucq et al. (2011), a primary reason most running wheel studies use no wheel access (as opposed to locked running wheels) as their control condition is the economic burden of commercial running wheels (Dubreucq et al., 2011). Although this is a practical concern, providing a locked running wheel better addresses potential environmental enrichment and novelty-related effects (Dubreucq et al., 2011). The cost-effectiveness of the DSC running wheel and their ability to lock ensures broad access to this important environmental control. However, a major limitation to the present work is the lack of sedentary (i.e., wheel-lock) comparisons. Ongoing studies are utilizing locked wheels for further behavioral testing in iHDID-1 and HS/Npt mice.

**Table 1:** Materials list for DSC-wheel and experimental set-up.

**Item**	**Manufacturer**	**Quantity**	Total cost (USD)^*^
**Major equipment**			
Creality ender 3 3D printer	Creality	1	$189.00
Mini PC (12 GB + 256 GB/Intel Celeron N5105)	KAMRUI	1	$169.99
Arduino UNO REV 3	Arduino	3	$86.97 ($28.99/unit)
Soldering station	TILSWALL	1	$74.99
PETG filament (1.75 mm, 1 kg)	OVERTURE	4	$67.96 ($16.99/unit)
Dremel 7760 lite rotary tool	Dremel	1	$64.00
Adafruit assembled data logging shield for Arduino	Adafruit	3	$41.85 ($13.95/unit)
Heat gun (1350 Watt)	BLACK + DECKER	1	$29.40
16 GB SD card (2 pcs)	PNY	2	$27.58 ($13.79/unit)
Arduino pin headers (112 pcs)	Glarks	1	$12.98
Self-adjusting wire stripper	Amazon basics	1	$9.99
Needle-nose pliers (6 in)	Outeels	1	$8.99
Brass extruder nozzles (0.2 mm, 0.3 mm, 0.4 mm, 0.5 mm, 0.6 mm, 0.8 mm, 1.0 mm- 24 pcs)	LUTER	1	$8.49
USB SD card reader	Vanja	1	$7.99
CR1220 3V Lithium coin cell battery (5 pack)	Toshiba	1	$5.95
Total major equipment cost:			$806.13
**Consumables**			
Plastic magnetic reed switch (14 mm- 10 pcs)	DIYhz	5	$49.95 ($9.99/unit)
Ball bearing (10 mm x 26 mm x 8 mm) double sealed chrome steel (10 pcs)	uxcell	5	$44.95 ($8.99/unit)
Pluggable LED wire connectors (20–24 AWG- 12 pcs)	Tyumen	5	$39.95 ($7.99/unit)
Electrical wire 24/2 AWG (32.8 ft)	TZMOIK	3	$29.97 ($9.99/unit)
Round magnets 6 mm x 2 mm (25 pcs)	NEXLEVL	4	$27.96 ($6.99/unit)
Tin lead rosin core solder wire (0.8 mm 50 g)	MAIYUM	2	$17.98 ($8.99/unit)
Jumper wires (120 pcs)	ELEGOO	1	$6.98
Super glue (0.07 oz/2g- 4 pcs)	The original super glue	1	$4.79
Scotch vinyl electrical tape (3/4 in x 66 ft)	Scotch	1	$2.78
Total consumables cost			$225.31
Total cost for DSC-wheels (50–60) and data loggers (3)			$1,031.44

^*^Cost as of September 24th, 2023- subject to change depending on time, economy, and location.

### 2.3 DSC-wheel assembly

To build a DSC-wheel, we first removed the 3D-printed supports on the bottom of the wheel and the base (using needle-nose pliers). Next, a rotary tool (Dremel 7760 Lite; Prospect, Illinois), with a 12.7 mm sanding drum, was used to expand the back center of the wheel to make space for the metal bearing. After the metal bearing was placed in the back of the wheel, we attached the axle to the base. To do this, we secured the axle to the base using one nut underneath the base and two nuts above the base, which acted as a jam nut. At this point, the wheel was added on top of the existing structure, and a final nut was added above the wheel (this nut was left partially unscrewed to allow for free spinning of wheel).

Hardwiring the wheels began with ~600 mm of 33 AWG lamp wire, where a wire stripper was used to remove 25.4 mm of plastic covering on one end, and 12.7 mm on the other. With the 12.7 mm of exposed wire, we used a soldering pen to solder each strand (copper or silver) to the two opposing charge terminals of the pluggable wire connector. We then threaded the available end of the wire through the axle and into the base and soldered the remaining 25.4 mm of exposed wire to both sides of the plastic magnetic reed switch (copper on one side, silver on the other). We were now able to pull the wire further up the axle, so the reed switch was nearly touching the second opening on the base. We then added a protective cap (attained by cutting 10 mL plastic serological pipette tips) over the reed switch and secured it within the base to both prevent chewing of equipment, and to have the reed switch in a vertical position to cleanly detect wheel rotations. To complete the DSC-wheel, we glued two stacked magnets (6 mm x 2 mm) near the back center of the wheel, so they crossed over the reed switch when the wheel was rotated. Once all DSC-wheels were completed, they were placed in the back right corner of each mouse cage, with ~50.8 mm of the axle sticking through the wire cage tops to prevent any chewing/ingestion of wire.

### 2.4 DSC-wheel code and analyses

DSC-wheel code was programmed in Arduino/C++ language using Arduino Integrated Development Environment (IDE; Arduino, New York, New York). Refer to the following link to access wheel-running analysis codes with annotation: https://github.com/grigsbkb/Dependable-Simple-and-Cost-effective-DSC-running-wheel. Briefly, up to 13 wheels were continuously queried by a single Arduino. If a given reed switch is observed to transition from open to closed (caused by the physical passing of the magnet on a rotating wheel) then a rotation is recorded for that wheel. Every minute the number of open events, close events, the total duration the switch spent open and closed, and the number of inferred rotations for each wheel is recorded as a .csv file on a local SD card. Note, any rotations observed to occur within 30 ms of a previously detected rotation were assumed to be caused by the physical bouncing of the reed switch and subsequently ignored. This debouncing delay is adjustable and the value chosen here of 30 ms was observed to be ~10x longer than the bouncing signal and would correspond to a wheel speed >55 km/h, much higher than the sprinting speed of mice. It should be noted that the Real-time Clock (RTC) feature on the Arduinos used in this study was not functioning. As a result, time was kept manually during the acute study, where we recorded what time Arduinos were reset every day for the duration of the experiment. Arduino reset times were not recorded during the first 4 days of the chronic study, so for the duration of this experiment, they were calculated as the average number of seconds taken (between each day) to reset Arduinos in the acute study.

### 2.5 Experiment 1: Acute wheel-running

To assess baseline voluntary running behaviors of male and female iHDID-1 and HS/Npt mice during acute wheel-running, mice (*n* = 8–10/sex/genotype) were given wheel access for 13 days, a time point where physical activity is considered physiologically stressful (Fediuc et al., 2006; Grigsby et al., 2022). On day 1 of the experiment, a DSC-wheel was placed in each mouse cage and remained there for the duration of the 13-day study (with the exception of a soiled, broken, or offline wheel, which was promptly replaced). Using pluggable connectors, lamp wire, and jumper wires (Figure 1), wheels were plugged in to their respective Arduino on day 1 to start recording data, and were labeled both by their mouse number and channel number (on Arduino; channels 0–1, 3–9, 14–17) for data analysis. Three Arduinos were used for all mouse studies, where Arduino number and channel number were used to identify mice for data collection and analysis. For the following 13 days, data was checked and saved every morning within 30-min of lights off ensure all wheels were functioning. To do this, SD cards were removed from each Arduino, inserted into a USB SD card reader to view data files on a PC (opened in.csv format), and daily data was saved as Excel files and stored on a server for subsequent analysis. After data collection on each day, Arduinos were reset for the following day. At the end of the study, mice were euthanized using carbon dioxide (CO_2_) followed by cervical dislocation. It should be noted that experiment 1 was originally intended to be a 14-day study. However, insufficient data was collected on day 14 of WR due to a building-wide generator test, so it has been removed from all analyses.

### 2.6 Experiment 2: Chronic wheel-running

To assess baseline differences in voluntary running behaviors of male and female iHDID-1 and HS/Npt mice during chronic wheel-running, all mice (*n* = 9–10/sex/genotype) were given wheel access for 4-weeks, a time-point where physical activity causes little to no stress response and is considered behaviorally rewarding (Greenwood et al., 2011; Grigsby et al., 2022). The procedure for wheel-running was carried out exactly as described above for experiment one (the only difference being a 28-day wheel-running period as opposed to a 14-day wheel-running period). At the end of the study, all mice were euthanized using CO_2_ followed by cervical dislocation.

The chronic WR study was conducted prior to the acute WR study and was the first undertaken using the DSC running wheels. Of note, wheels would sometimes go offline due to a broken/non-functioning glass reed switch, which appeared sufficient in preliminary studies but proved to be far too fragile at large scale. Although these wheels were promptly replaced the following day, these technical issues resulted in missing or suppressed data during the first 2-weeks of data acquisition. We present all four weeks of data in this manuscript to show the ability of our running wheels to be used in a longer-term study (with appropriate adaptations to wheel design). Notably, glass reed switches were replaced with plastic reed switches (included in Table 1 materials), which ensured nearly all wheels staying online for the last 2-weeks of experiment 2 and the entire duration of experiment 1.

### 2.7 Statistics

**Figure 2:**
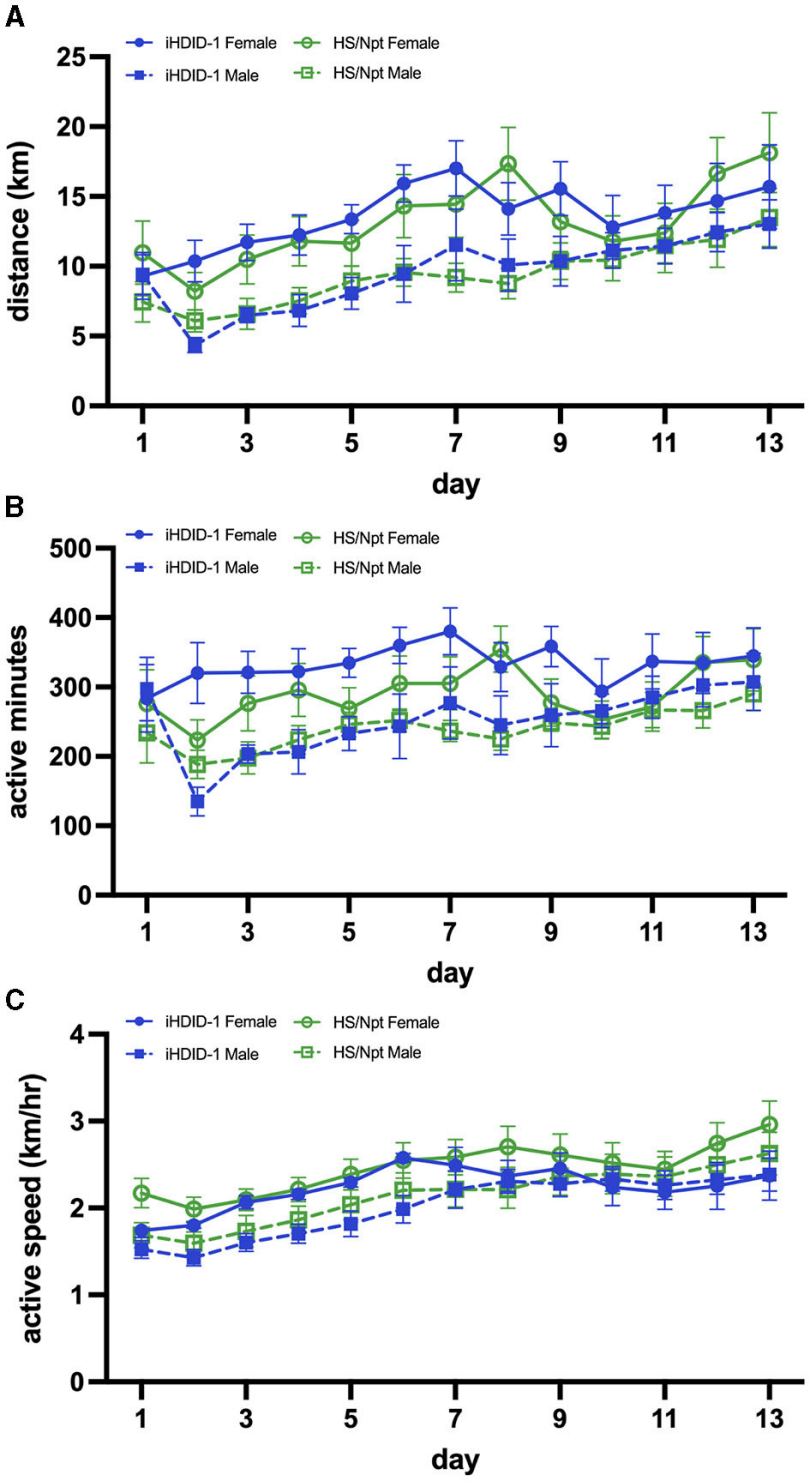
**(A)** Average daily WR distance (km) for HS/Npt and iHDID-1 mice (*n* = 8–10/sex/genotype) during acute WR (weekly statistics are reported); main effect of week [F(1,34) = 31.17; *p* < 0.0001] and sex [F(1,35) = 6.29; *p* < 0.05]. **(B)** Average daily WR duration (active minutes; i.e., > 30 wheel rotations in 1 min- refer to Section 2.7 of methods for justification) for HS/Npt and iHDID-1 mice during acute WR (weekly statistics are reported); main effect of week [F(1,34) = 8.78; *p* < 0.01] and sex [F(1,35) = 6.16; *p* < 0.05]. **(C)** Average daily WR speed (km/hr) for HS/Npt and iHDID-1 mice during acute WR (weekly statistics are reported); main effect of week [F(1,36) = 64.20; *p* < 0.0001].

All statistical analyses of mouse studies were conducted with GraphPad Prism Software Version 9.0 and R. The sample sizes for each experiment are reported in the appropriate figure legend. If no significant main or interaction effects of sex were observed, we performed statistical analyses on data collapsed across sexes. Data are presented as mean +/- SEM. Average weekly distance (km/week), running duration (active minutes/day; which was defined as > 30 wheel rotations/minute for acute WR and > 17 wheel rotations/minute for chronic WR [lower quartile value of wheel rotations/minute]), and speed (only during active minutes; km/hour) for this study were analyzed using a three-way repeated measures ANOVA [sex x genotype x time (week)] in GraphPad. To better visualize running characteristics at acute and chronic time-points, daily running averages are presented in Figures 2, **5**. When data points were missing (e.g., if a wheel was broken or offline), a mixed-effects three-way RM ANOVA was employed. It should be noted that if a mouse did not run for at least 20 active minutes in one day, its data from that day was excluded from statistical analyses. This cutoff was implemented to minimize the inclusion of data from broken/offline wheels, and to reflect adult human PA guidelines more accurately (~20–40 min/day) (US Department of Health and Human Services, 2018). The significance cutoff for all statistical analyses in this study was p < 0.05. Additionally, data for mouse 20 (HS/Npt male) was excluded on days 13 and 23 of chronic WR due to extreme speed values (km/hr) that were much higher than the speed of this mouse on other days, all other mice in experiment 2 (Supplementary Figure 2C; mouse 20 is labeled as MouseID 3_6), and speeds previously reported in C57BL/6J mice (Manzanares et al., 2018) (indicating a malfunction in equipment, as opposed to true speed).

## 3 Results

### 3.1 Experiment 1: Acute (13-day) wheel-running behavior depends on time and sex, but not genotype

To examine wheel-running behaviors in male and female iHDID-1 and HS/Npt mice during acute wheel-running, mice (*n* = 8–10/sex/genotype) were given access to an open running wheel for 14-days. A mixed-effects analysis was used to analyze weekly averages of running distance (km/day), duration (active minutes/day), and speed (km/hour). Analysis of average weekly running distance (km) revealed a main effect of week [F(1,34) = 31.17; *p* < 0.0001] and sex [F(1,35) = 6.29; *p* < 0.05] (Figure 2A). Analysis of average weekly running duration (active minutes/day) revealed a main effect of week [F(1,34) = 8.78; *p* < 0.01] and sex [F(1,35) = 6.16; *p* < 0.05] (Figure 2B). Analysis of average weekly running active speed (km/hr) revealed a main effect of week [F(1,36) = 64.20; *p* < 0.0001] (Figure 2C). No main effects of genotype or genotype-interactions were present for average weekly running distance, duration, or speed.

### 3.2 Experiment 1: 24-h activity patterns of male and female iHDID-1 and HS/Npt mice during acute wheel-running

**Figure 3:**
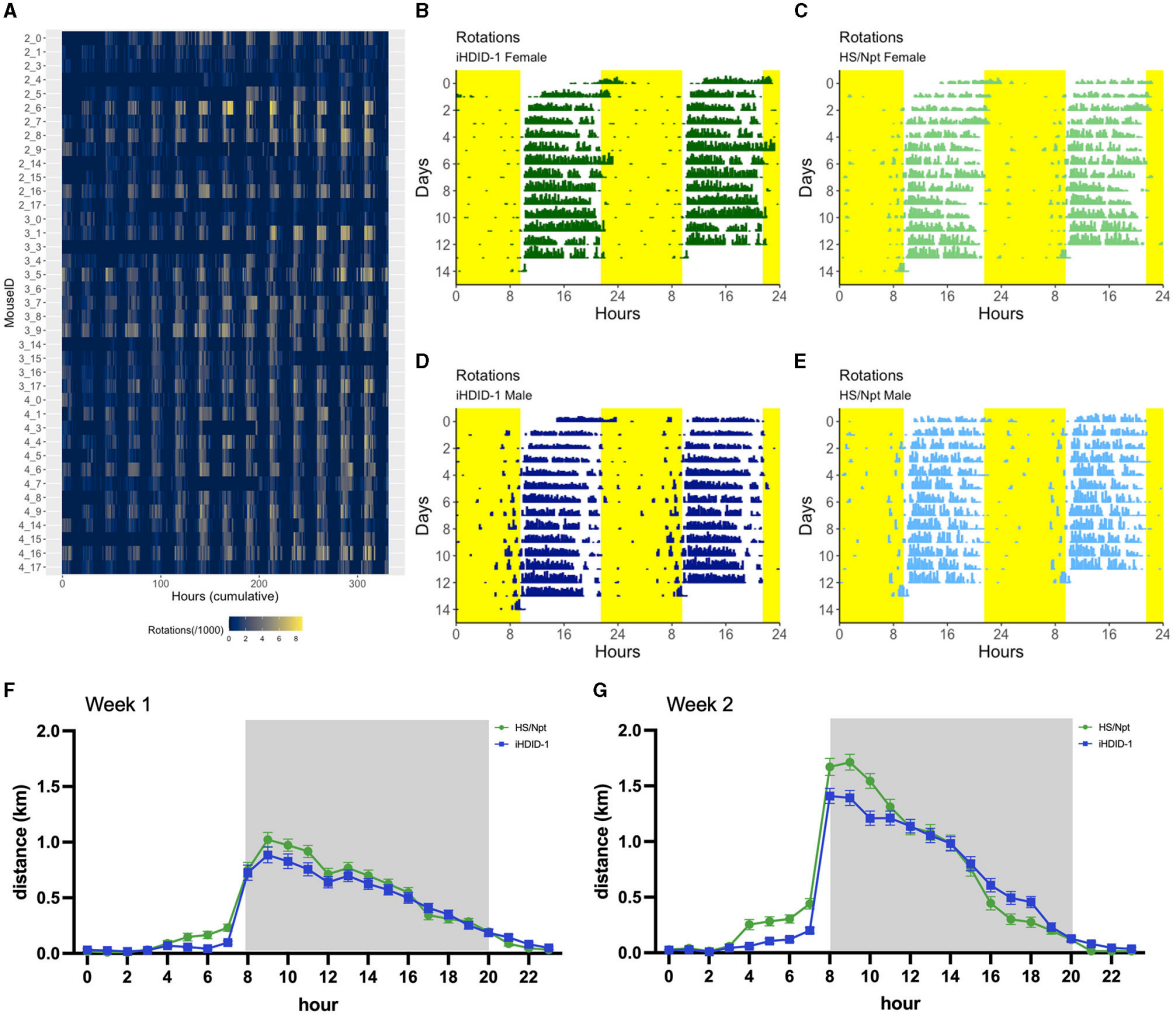
**(A)** Heatmap displaying wheel rotations/hour (grouped in 10-min bins) throughout a 13-day (acute) wheel-running study for all mice (*n* = 8–10/sex/genotype). The x-axis shows hours (ranging from the beginning of the study to the end), and the y-axis shows individual MouseID (a unique identifier obtained from the Arduino number and channel number each mouse wheel was connected to; format: Arduino number_channel number). Each bar on the heatmap represents wheel rotations/10 min bin, where the number of rotations occurring is proportional to the color of the bar. **(B, C)** Double-plotted actograms displaying wheel rotations over a 24-h time period (grouped in 10-min bins) throughout a 13-day wheel-running study from one representative iHDID-1 Female (MouseID: 4_1) **(B)** and HS/Npt Female **(**MouseID: 3_17) **(C)**. **(D, E)** Double-plotted circadian actograms displaying wheel rotations over a 24-h time period (grouped in 10-min bins) throughout a 13-day wheel-running study from one representative iHDID-1 Male (MouseID: 3_8) **(D)** and HS/Npt Male (MouseID: 4_9) **(E)**. Yellow areas note 12-h blocks where lights were on during a 12-h light-dark cycle. The x-axis shows the time of day (where the plot repeats starting at 24-h), and the y-axis shows the total number of days in the study. Each bar on the actogram represents wheel rotations/10 min bin, where the number of rotations occurring is proportional to the height of the bar. **(F)** Average hourly running distance (km/hr) during week 1 of acute WR, where the gray area denotes the 12-h dark period of the light/dark cycle. **(G)** Average hourly running distance (km/hr) during week 2 of acute WR, where the gray area denotes the 12-h dark period of the light/dark cycle.

To visualize wheel-running activity patterns over a 24-h period for 13-days, we created double-plotted actograms for one representative study mouse per experimental group; iHDID-1 female (Figure 3B), HS/Npt female (Figure 3C), iHDID-1 male (Figure 3D), and HS/Npt male (Figure 3E) using R (see Section 2.4 of methods for GitHub link to acute WR analysis code). Representative mice were chosen by consistency of activity throughout the experiment (MouseID: 4_1 [iHDID-1 F], 3_17 [HS/Npt F], 3_8 [iHDID-1 M], and 4_9 [HS/Npt M]; (Figure 3A)]. The granular wheel-running analysis code created in this study allowed us to measure and visualize circadian activity patterns in 10-min bins. 24-h activity is comparable among genotype and sex, with wheel-running (rotations/10-min bin) occurring mainly during the dark period of a 12-h light-dark cycle.

McCulley et al. found distinct genotype differences in daily activity patterns between male HDID-1 and HS/Npt mice under standard 12 h:12 h conditions, whereby HDID-1 showed lower activity than HS/Npt during the early hours of the dark cycle (~2–6 h after lights-off) and greater activity in the late dark phase (~2 h before lights on) and early light phase (~2–3 h after lights on) (McCulley et al., 2013). To determine whether iHDID-1 mice maintain similar activity patterns, we compared 24-h running distance (km/hr) between female and male iHDID-1 and HS/Npt for experiment 1 (Figures 3F, G). There was no main effect of sex on average running distance for weeks 1 and 2; therefore, data has been collapsed on sex. A mixed-effects analysis of average hourly running distance (km/hr) revealed main effects of hour [F(23, 38) = 140.3; *p* < 0.0001] and genotype [F(1,38) = 12.63; *p* < 0.001], with no hour x genotype interaction. Post-hoc testing revealed that iHDID-1 ran significantly less than HS/Npt at hour 11 in week 1 (Figure 3F) and hours 8–10 in week 2 (Figure 3G), and significantly more than HS/Npt in hours 16,17, and 18 of week 2. To better visualize daily activity pattern characteristics, the 24-h running distance (km/hr) of iHDID-1 and HS/Npt are presented in quartiles [High (upper quartile); Average (middle quartiles); and Low (lower quartile)] for experiments 1 (Supplementary Figure 3A)**]** and 2 (Supplementary Figure 3B)**]**.

### 3.3 Experiment 1: Distribution of wheel rotations in male and female iHDID-1 and HS/Npt mice on day 1 and day 13 of acute wheel-running

**Figure 4:**
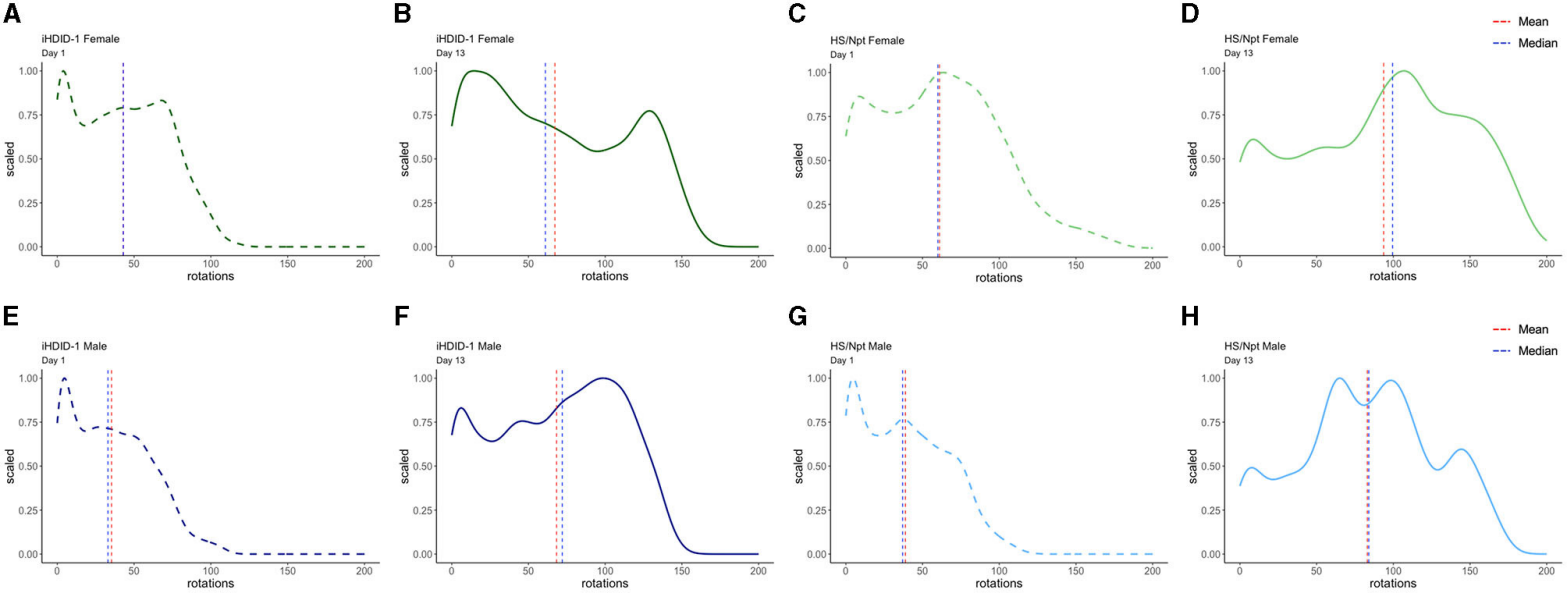
Density plots of wheel rotations for every minute with more than zero rotations on days 1 (dashed) and 13 (solid) of acute WR. In each plot, the vertical red line represents the mean value in each distribution, and the vertical blue line represents the median value in each distribution. The x-axis shows the range of wheel rotations in the acute WR dataset and the y-axis shows scale (representing the frequency of wheel rotations occurring on a 0–1 scale). **(A, B)** Density plots for iHDID-1 female wheel rotations on day 1 and day 13 of acute WR. **(C, D)** Density plots for HS/Npt female wheel rotations on day 1 and day 13 of acute WR. **(E, F)** Density plots for iHDID-1 male wheel rotations on day 1 and day 13 of acute WR. **(G, H)** Density plots for HS/Npt male wheel rotations on day 1 and day 13 of acute WR.

**Figure 5:**
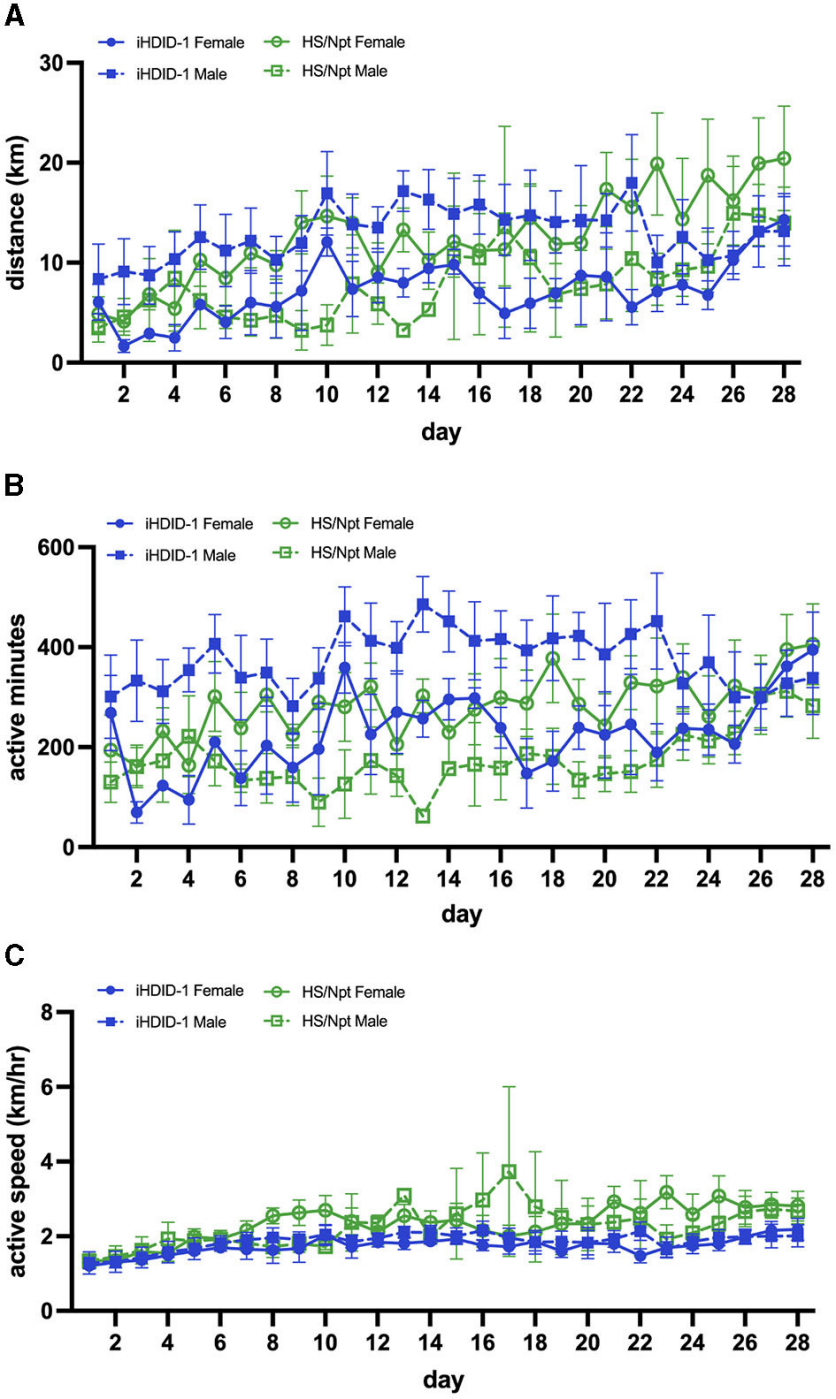
**(A)** Average daily WR distance (km) for HS/Npt and iHDID-1 mice (*n* = 5–10/sex/genotype) during chronic WR (weekly statistics are reported); main effect of week [F(2.69,67.25) = 7.42; *p* < 0.001], and a sex x genotype interaction [F(1,34) = 5.75; *p* < 0.05]. A post-hoc Sidak test revealed no significant effects. **(B)** Average daily WR duration (active minutes; i.e., > 17 wheel rotations in 1 min- refer to Section 2.7 of methods for justification) for HS/Npt and iHDID-1 mice during chronic WR (weekly statistics are reported); main effect of week [F(2.64,66.08) = 3.13; *p* < 0.05], genotype [F(1,34) = 12.67; p < 0.01], and a sex x genotype interaction [F(1, 34) = 9.32; *p* < 0.01]. A post-hoc Sidak test revealed no significant effects. **(C)** Average daily WR active speed (km/hr) for HS/Npt and iHDID-1 mice during chronic WR (weekly statistics are reported); main effect of week [F(1.94, 52.39 = 8.52); *p* < 0.001].

To address WR pattern differences in male and female iHDID-1 and HS/Npt mice on the first and last day of acute WR, density plots were generated using R. Density plots allow us to visualize the distribution of wheel rotations using a continuous curve. This also makes it simpler to identify distribution shape and overall shifts in data. Figure 4 shows density plots for wheel rotations on day 1 and day 13 of acute WR for iHDID-1 female mice (Figures 4A, B), HS/Npt female mice (Figures 4C, D), iHDID-1 male mice (Figures 4E, F), and HS/Npt male mice (Figures 4G, H). Mean rotations are right shifted on day 13 of acute WR, as compared to day 1, showing how mice (of both sexes and genotypes) change their WR behavior over a 2-week period. Minutes with zero rotations were excluded from density plots. This cutoff was implemented because the majority of minutes during the acute WR experiment had zero rotations for every wheel (76%), and for the purposes of this study, we are only interested in minutes where mice are active to elucidate their running patterns between groups and across time. Additionally, we did not apply the same data filtering we used for Figures 2, 5 (see Section 2.7 of methods) here because we are filtering rotations by minutes (on day 1 and day 13) as opposed to filtering by days.

### 3.4 Experiment 2: Chronic (28-day) wheel-running behavior depends on time, sex, and genotype

To examine wheel-running behaviors in male and female iHDID-1 and HS/Npt mice at a time point where PA is considered behaviorally reinforcing, mice (*n* = 8–10/sex/genotype) were given access to an open running wheel for 28-days. A mixed-effects analysis was used to analyze average weekly running distance (km/day), duration (active minutes/day), and speed (km/hour). Analysis of average weekly running distance (km) revealed a main effect of week [F(2.69,67.25) = 7.42; *p* < 0.001], and a sex x genotype interaction [F(1,34) = 5.75; *p* < 0.05]. A post-hoc Sidak test revealed no significant group differences (Figure 5A). Analysis of average daily running duration (active minutes) revealed a main effect of week [F(2.64,66.08) = 3.13; *p* < 0.05], genotype [F(1,34) = 12.67; p < 0.01], and a sex x genotype interaction [F(1, 34) = 9.32; *p* < 0.01]. A post-hoc Sidak test revealed no significant differences (Figure 5B). Analysis of average weekly running speed (km/hr) revealed a main effect of week [F(1.94, 52.39 = 8.52); *p* < 0.001] (Figure 5C).

### 3.5 Experiment 2: Distribution of wheel rotations in male and female iHDID-1 and HS/Npt mice on day 1 and day 28 of chronic wheel-running

**Figure 6:**
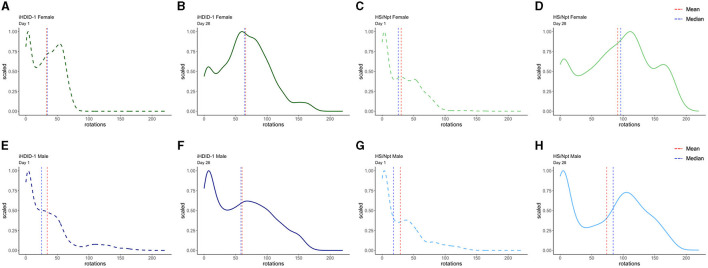
Density plots of wheel rotations for every minute with more than zero rotations on days 1 (dashed) and 28 (solid) of chronic WR. In each plot, the vertical red line represents the mean value in each distribution, and the vertical blue line represents the median value in each distribution. The x-axis shows the range of wheel rotations in the chronic WR dataset and the y-axis shows scale (representing the frequency of wheel rotations occurring on a 0–1 scale). **(A, B)** Density plot for iHDID-1 female wheel rotations on day 1 and day 28 of chronic WR. **(C, D)** Density plot for HS/Npt female wheel rotations on day 1 and day 28 of chronic WR. **(E, F)** Density plot for iHDID-1 male wheel rotations on day 1 and day 28 of chronic WR. **(G, H)** Density plot for HS/Npt male wheel rotations on day 1 and day 28 of chronic WR.

To visualize WR pattern differences in male and female iHDID-1 and HS/Npt mice on the first and last day of chronic WR, density plots were generated using R. Figure 6 shows density plots for wheel rotations on day 1 and day 28 of chronic WR for iHDID-1 female mice (Figures 6A, B), HS/Npt female mice (Figures 6C, D), iHDID-1 male mice (Figures 6E, F), and HS/Npt male mice (Figures 6G, H). Mean rotations are right shifted on day 28 of chronic WR, as compared to day 1, showing how mice (of both sexes and genotypes) change their WR behavior over a 4-week period. Minutes with zero rotations were excluded from density plots. This cutoff was implemented because the majority of minutes during the chronic WR experiment had zero rotations for every wheel (85%), and for the purposes of this study, we are only interested in minutes where mice are active to elucidate their running patterns between groups and across time. Additionally, we did not apply the same data filtering we used for Figures 2, 5 (see Section 2.7 of methods) here because we are filtering rotations by minutes (on day 1 and day 28) as opposed to filtering by days.

## 4 Discussion

The present findings describe the development and testing of an open-source, 3D printable running wheel design by characterizing running patterns in both genetically heterogenous mice and a genetic risk model for drinking to intoxication. The DSC-wheel provides highly granular and customizable data acquisition at an extremely low price point. Using these wheels, we were able to characterize 24-h running activity (i.e., actograms), distribution of wheel rotations, as well as running distance, duration, and speed at two critical stages of PA development and reinforcement. We found that both HS/Npt and iHDID-1 mice exhibited similar running patterns compared to other inbred strains (including 7 out of the 8 inbred strains that comprise the HS/Npt founder line; Lightfoot et al., 2004). Collectively, our data demonstrates the ability of these low-cost, high throughput DSC-wheels to collect accurate and robust data in real time. The DSC-wheel is accessible and can be used in research efforts spanning the biomedical field, including research on circadian activity, motivation, and the therapeutic potential of PA to treat diseases and disorders, including harmful substance and alcohol use.

Experiment 1 of the present study characterized the wheel-running behaviors of female and male HS/Npt and iHDID-1 mice during acute (13-days) WR. Overall, the present findings are in alignment with past work showing that WR increases over time, and that female rodents tend to run more than male rodents (Eikelboom and Mills, 1988; Cordony et al., 2019). We also generated density plots for days 1 and 13 to address the influence of day and sex during early stages of PA reinforcement. We did not observe any striking strain or sex-specific differences in running distribution, with all groups of mice displaying a left skew in their running patterns on day 1 of acute WR and trending toward a more normal distribution by day 13. This suggests that, on average, mice tend to travel shorter distances (at a slower pace) in week 1 and run further (at a faster pace) by the end of week 2. This reflects past literature showing the acquisition of WR in female Sprague Dawley rats, where running distances were shown to escalate during the first 2 weeks of access (Cordony et al., 2019). Through characterizing the distribution in running patterns over time (particularly in heterogenous mice, like the HS/Npt), future research can help identify individual differences in the escalation and reinforcement of PA and better inform the use of PA to treat other, more harmful patterned behaviors.

Acute PA, like other behavioral coping strategies (i.e., alcohol and substance use), increases physiological markers of stress. Work by Fediuc et al. found that 1- to 2-week of wheel-running increases plasma corticosterone levels in male Sprague Dawley rats, whereas 4-weeks of wheel access evokes little to no stress response (Fediuc et al., 2006). Similarly, 1-week of running was shown to increase corticosterone levels and adrenal wet weights in male low voluntary running rats (compared to no running and 4-week running conditions), as well as elevate basolateral amygdala levels of prodynorphin and Kappa Opioid Receptor (OPRK1) phosphorylation, both of which are markers of aversion-related signaling (Grigsby et al., 2022). To our knowledge, a parallel study addressing the sex-specific effects of acute and chronic WR on markers of Hypothalamic Pituitary Adrenal (HPA)-axis responsiveness (i.e., plasma corticosterone and adrenal wet weights) has not been published. This is especially important given that female rodents tend to run greater distances and faster speeds than their male counterparts (Eikelboom and Mills, 1988; Lightfoot et al., 2004), have higher baseline corticosterone levels, and have higher corticosterone responses to ethanol injections than males (Lightfoot et al., 2004; Willey et al., 2012; Bangasser and Wicks, 2017). Moreover, recent work from Savarese et al. found that female and male iHDID-1 mice have higher corticosterone responses to a mild stressor (i.p. injections of ethanol or saline) than HS/Npt mice and show reduced glucocorticoid receptor (GR) gene expression in the nucleus accumbens (NAc; critical role in reinforcing and motivated behaviors) both basally and following ethanol administration (2 g/kg) (Savarese et al., 2022). Future work using these mice would help identify potential neurobiological markers of stress-related signaling (i.e. GR expression) in response to WR and better elucidate the role of stress in the reinforcement of PA.

Harmful ethanol consumption is known to alter circadian activity in rats and mice (Rosenwasser et al., 2005a,c; Seggio et al., 2009). Like HDID mice, High Alcohol Drinking/Preferring rats show different circadian activity patterns than Low Alcohol Drinking/Preferring rats, indicating that selection for ethanol-related phenotypes may alter circadian-related behaviors in rodents- likely through a shared genetic relationship (Hofstetter et al., 2003; Rosenwasser et al., 2005b). As highlighted in Section 3.4 of results, the DSC-wheel can capture 24-h running data on a granular scale, which has broad applications for the circadian and addiction fields. Historically, circadian studies have required the use of expensive equipment and/or software to measure 24-h activity patterns (Siepka and Takahashi, 2005). We created representative actograms (for each sex and strain) based on acute wheel-running data using R. Previous work found genotype differences in daily running activity between HDID and HS/Npt mice during the dark phase and early light phase of their light/dark cycle. The present findings suggest that female and male iHDID-1 maintain similar daily running patterns, whereby they tend to run less than HS/Npt in the early hours of the dark phase and more than HS/Npt in the later hours of the dark cycle. Although we saw no genotype differences in the light phase for experiment 1, genotype comparisons of 24-h activity patterns in experiment 2 (Supplementary Figure 3B) show that iHDID-1 are more active than HS/Npt in the early hours of the light phase in weeks 2 and 3. In support of existing binge-like and aversion resistant drinking data, it appears that iHDID-1 similarly maintain the daily activity patterns of HDID-1. McCulley et al. also found that HDID mice show shorter free-running circadian periods than HS/Npt mice under constant light conditions (McCulley et al., 2013). While free running circadian rhythms were not tested in the present work, we aim to implement the DSC-wheel in future studies comparing circadian running patterns of male and female iHDID-1 and HS/Npt mice at acute and chronic timepoints. Future work will include testing the effects of light/dark manipulations on running patterns in male and female iHDID-1 and HS/Npt mice in the context of binge-like ethanol drinking.

Experiment 2 of the present study characterized the wheel-running behaviors of female and male HS/Npt and iHDID-1 mice during chronic (28-days) of WR. It is important to note that there were many technical issues during data acquisition for weeks 1 and 2 of this study and therefore the following interpretations should be taken with reservations. Compared to acute WR, density plots generated for the last day of chronic WR trended toward a normal distribution and a right shift in running patterns, where mice ran further and at a faster pace at day 28 compared to day 1. This reflects previous work showing that male rats run for ~1 h following 1–2 weeks of WR and for ~9-h following 6-weeks of WR (Greenwood et al., 2011). Interestingly, mean rotations on day 28 are comparable to those on day 13 for all groups, suggesting that WR patterns are maintained following the 2-week timepoint. This observation is supported by the WR literature, which shows that rodent wheel-running increases during the first 1–2 weeks (acquisition phase), and shifts toward becoming a habitual behavior thereafter (maintenance phase) (Cordony et al., 2019; Greenwood and Fleshner, 2019). Density plots from both acute and chronic WR display running behaviors similar to those recorded in the WR literature, further emphasizing that our low-cost, open-source running wheels function similarly to commercial models (Greenwood et al., 2011; Cordony et al., 2019).

Running duration may provide a more translatable measure of PA reinforcement than distance or speed on the basis that U.S. guidelines advise ~20–40 min of moderate PA/day for adults (US Department of Health and Human Services, 2018). While both strains exceed this daily goal, iHDID-1 mice surpass HS/Npt mice in the time they spend engaged in running. HDID-1 mice have known differences in their binge-like drinking microstructure compared to HS/Npt mice, whereby they drink in larger bout volumes (Barkley-Levenson and Crabbe, 2015). Although the intake characteristics of iHDID-1 have not been directly tested, it follows that a maintained “gulping-like phenotype” may translate to alterations in other patterned behaviors (i.e., wheel-running). However, further behavioral testing is needed to measure the reinforcement and PA bouts of WR for these two strains.

In short, the DSC-wheel accurately captures wheel-running patterns on par with commercially available options. However, there are improvements that can be made to the design that may benefit data acquisition in future WR studies. It has been previously reported that rodents prefer running on a low-profile, disc-shaped wheel as compared to an upright wheel (Walker and Mason, 2018). Because the DSC-wheel utilizes a similar low-profile design to accommodate for this preference, it often collected waste (i.e., fecal matter), making the wheel more difficult to rotate. Wheels were replaced promptly and cleaned thoroughly, but to avoid waste collecting on the wheel in future studies, we plan to incorporate a mesh or honeycomb pattern (which would also reduce the 3D print time). Mice have been shown to run more on mesh flooring compared to parallel rods (Banjanin and Mrosovsky, 2000). Therefore, incorporating a mesh or honeycomb wheel pattern in the current DSC-wheel design will reduce waste build-up and may encourage voluntary WR. The axle, which connects the base and wheel (refer to Figure 1), was susceptible to breaking under pressure (i.e., during the assembly process or during cleaning). One way to mitigate this in future iterations is to include 3D-printed, x-shaped supports throughout the interior of the axle. A major limitation in our work includes the lack of data collected during the first 2-weeks of chronic WR due to broken or malfunctioning glass reed switches (refer to Section 2.6 of methods). While this issue was fully remedied by switching to plastic reed switches during the last 2-weeks of the chronic study and in the subsequent acute WR study, another option is to use a hall effect sensor. Hall effect sensors are smaller, more compact, do not rely on an internal physical mechanism, and have recorded data accurately and efficiently in other running wheel models (Bivona and Poynter, 2021; Edwards et al., 2021). We hope that making the aforementioned adaptations in wheel design will help refine the DSC-wheel and support optimal data acquisition.

Overall, these findings establish the utility and accessibility of the DSC running wheel by characterizing acute and chronic WR behaviors in a mouse line of risk for binge-like ethanol drinking and their heterogeneous founders. Future directions include investigating the effects of acute and chronic PA on binge-like ethanol drinking in iHDID-1 mice to better understand the role of PA in treating AUD.

## Data availability statement

The raw data supporting the conclusions of this article will be made available by the authors, without undue reservation.

## Ethics statement

The animal study was approved by VA Portland Health Care System Animal Care and Use Committee. The study was conducted in accordance with the local legislation and institutional requirements.

## Author contributions

KG: Conceptualization, Data curation, Formal analysis, Funding acquisition, Methodology, Project administration, Resources, Supervision, Writing—original draft, Writing—review & editing. ZU: Conceptualization, Data curation, Formal analysis, Investigation, Methodology, Validation, Visualization, Writing—original draft, Writing—review & editing. JA: Conceptualization, Data curation, Formal analysis, Methodology, Software, Validation, Visualization, Writing—original draft, Writing—review & editing. AO: Funding acquisition, Investigation, Project administration, Supervision, Writing—original draft, Writing—review & editing.
