# Supplementary material for: Development and implementation of a Dependable, Simple, and Cost-effective (DSC), open-source running wheel in High Drinking in the Dark and Heterogeneous Stock/Northport mice
Source: Front Behav Neurosci. 2024 Jan 15;17:1321349. doi: 10.3389/fnbeh.2023.1321349 (PMC10823001; doi:10.3389/fnbeh.2023.1321349)
Supplement: Supplementary file 1 [file Data_Sheet_1.docx]

Supplementary Material

Development and Implementation of a Dependable, Simple, and Cost-effective (DSC), Open-source Running Wheel in High Drinking in the Dark and Heterogeneous Stock/Northport mice

Kolter Grigsby^1,2†*^, Zaynah Usmani^1,2†^, Justin Anderson^1,2^, Angela Ozburn^1,2^

**^†^**These authors contributed equally to this work and share first authorship

^1^ Portland Veterans Affairs Medical Center, Research and Development Service, Portland, OR, USA 97239

^2^ Oregon Health and Science University, Department of Behavioral Neuroscience, Portland, OR, USA 97239

*** Correspondence:** Kolter Grigsby; grigsbyk@ohsu.edu

# Supplementary Figures


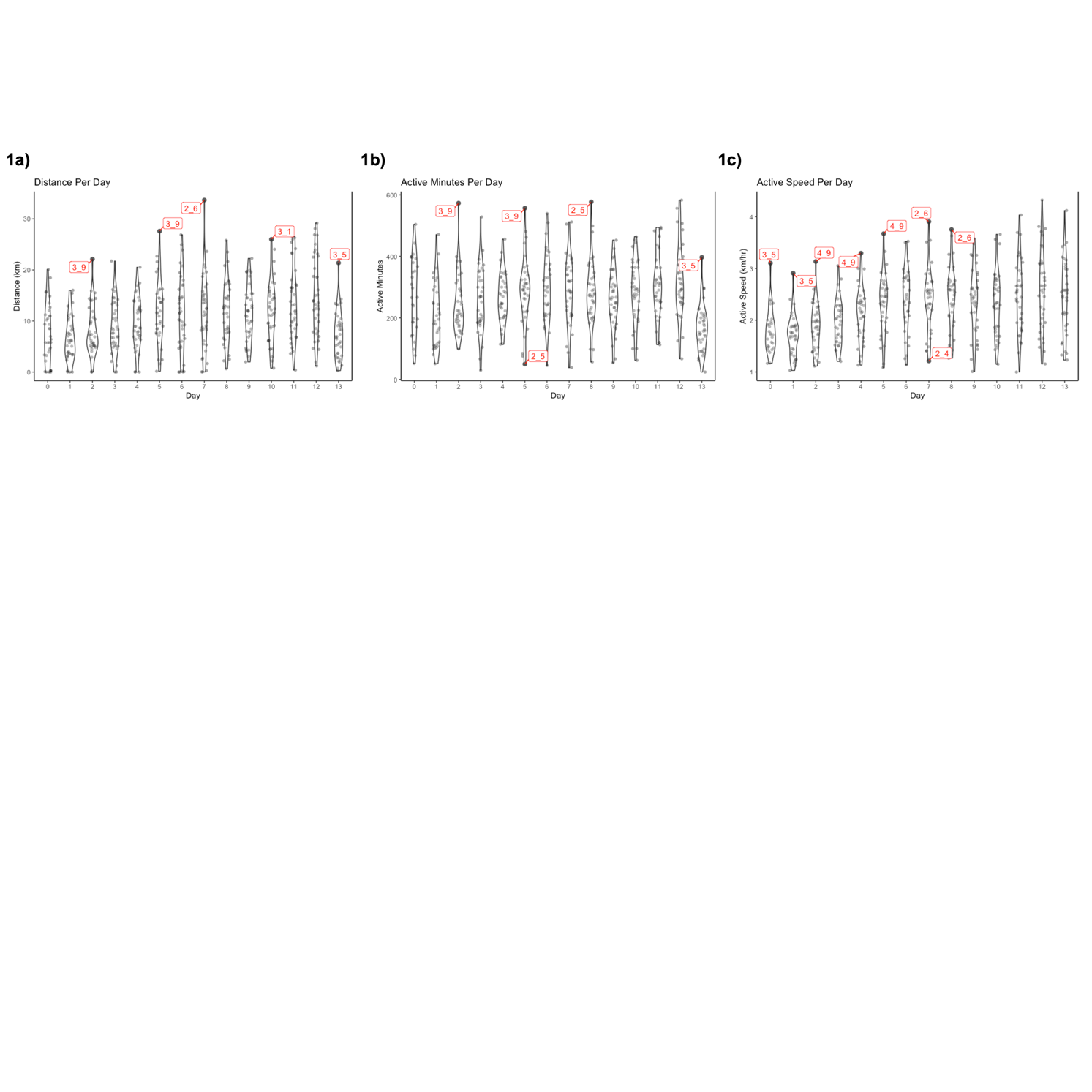
**Supplementary Figure 1.** Violin plots showing distribution of daily running distance, duration, and speed over the course of the acute WR study. In each plot, MouseIDs tagged in red indicate mice that ran outside of 1.5 times the inter-quartile range on that day (because our data is not distributed normally, we cannot identify these mice as outliers). **[A]** Violin plot showing distribution of WR distance (km) over 13-days of acute WR. **[B]** Violin plot showing distribution of WR duration (active minutes) over 13-days of acute WR. **[C]** Violin plot showing distribution of WR active speed (km/hr) over 13-days of acute WR.


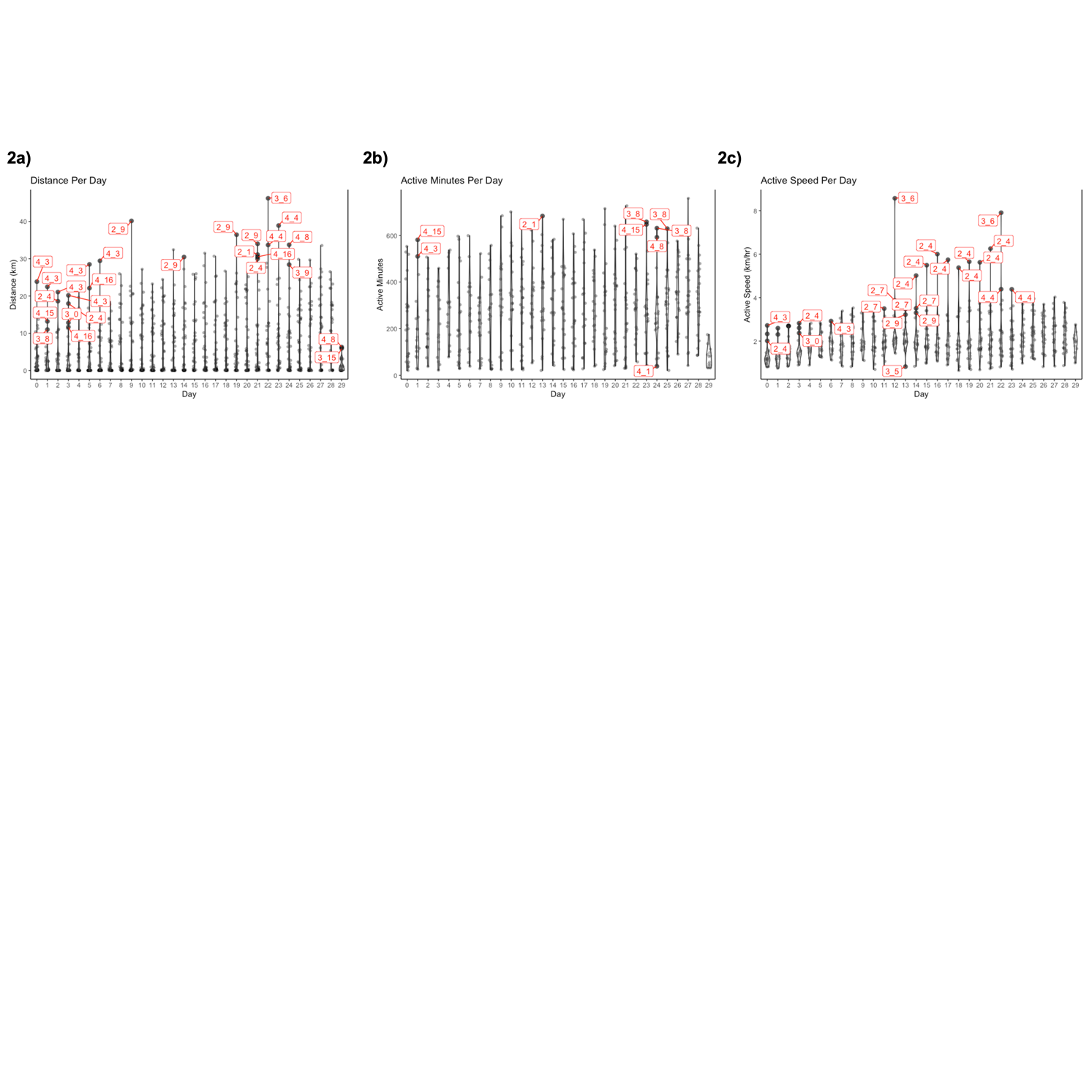
**Supplementary Figure 2.** Violin plots showing distribution of daily running distance, duration, and speed over the course of the chronic WR study. In each plot, MouseIDs tagged in red indicate mice that ran outside of 1.5 times the inter-quartile range on that day (because our data is not distributed normally, we cannot identify these mice as outliers). **[A]** Violin plot showing distribution of WR distance (km) over 28-days of chronic WR. **[B]** Violin plot showing distribution of WR duration (active minutes) over 28-days of chronic WR. **[C]** Violin plot showing distribution of WR active speed (km/hr) over 28-days of chronic WR.


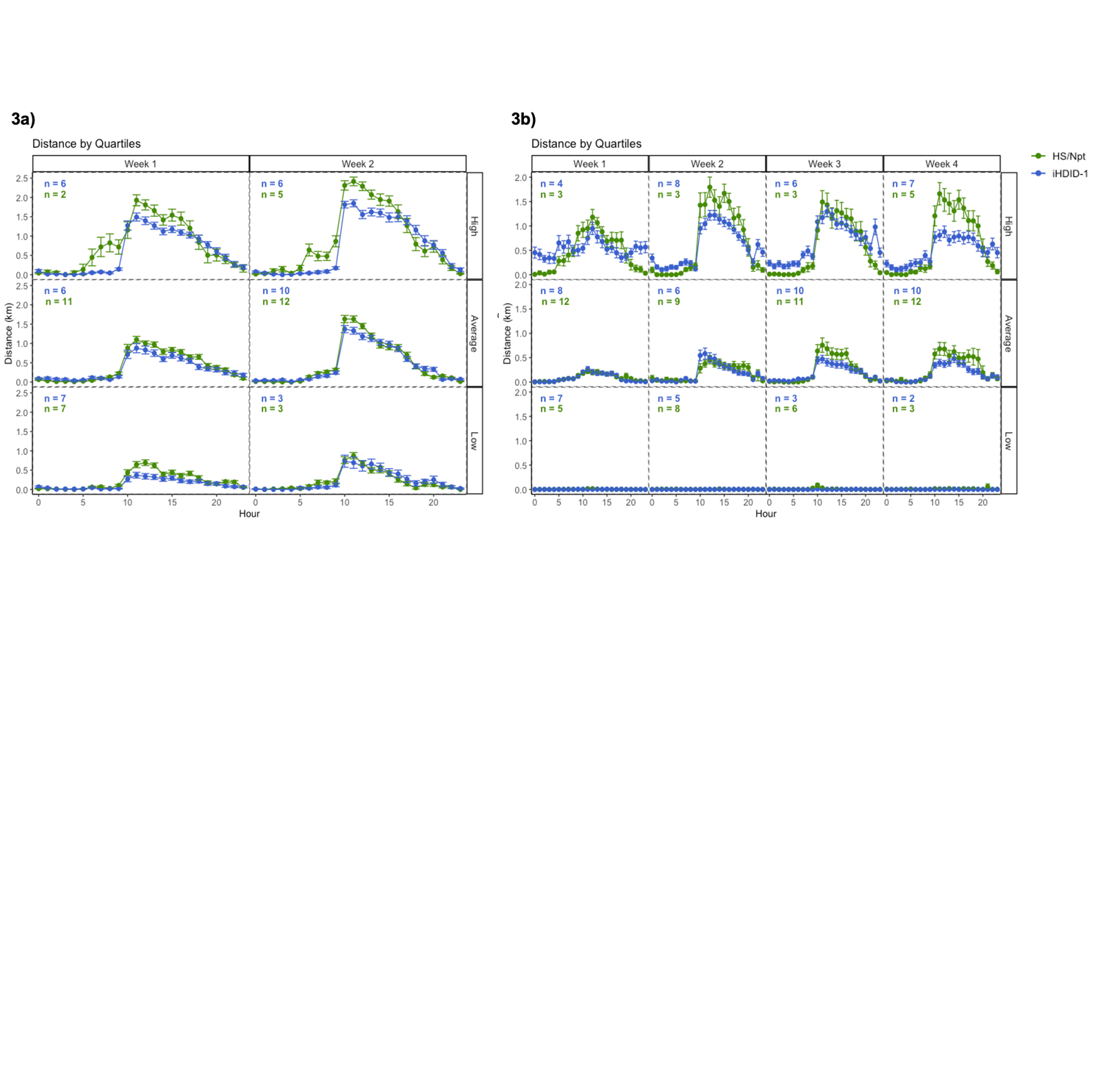
**Supplementary Figure 3.** 24-hour activity plots visualizing running distance (km/hr) of iHDID-1 and HS/Npt mice during **[A]** acute WR and **[B]** chronic WR. Hourly running data of both strains are presented in quartiles (High [upper quartile], Average [middle quartiles], and Low [lower quartile]).
